# Cognitive assessment tools for older adults in the Arabic language: a systematic review

**DOI:** 10.1002/alz.087484

**Published:** 2025-01-09

**Authors:** Mayssan Kabalan, Ola Bazzi, Josleen Al‐Barathie, Ola El Zein, Lara Chehabeddine, Monique Chaaya, Carlos Mendes de Leon, Martine Elbejjani

**Affiliations:** ^1^ Faculty of Health Sciences, American University of Beirut, Beirut Lebanon; ^2^ Saab Medical Library, American University of Beirut, Beirut Lebanon; ^3^ Georgetown University, Washington DC, DC USA; ^4^ Faculty of Medicine, American University of Beirut, Beirut Lebanon

## Abstract

**Background:**

Across the Arab world, Alzheimer’s disease and related dementias (ADRD) are a growing public health concern with the increase in the proportion of older adults. Yet, our understanding of these conditions remains limited because of a lack of data. Robust assessments of cognitive function are crucial for screening, prevention, and risk factor identification. This systematic review aims to identify available cognitive assessments in Arabic and assess their validity and performance.

**Method:**

We conducted a comprehensive search with a broad inclusion criterion using Medline OVID, EMBASE, and APA PsychInfo up to November 2023. Our search included both studies developing or validating cognitive assessment tools in Arabic for individuals aged 50 and above, and studies applying any such tool in the Arabic language. Study selection and data abstraction were carried out in duplicate and independently and using standardized and pilot‐tested forms. We analyzed and reported the availability, appropriateness, and use of the available cognitive assessment instruments.

**Result:**

Our review yielded 154 studies, of which 27 studies involved developing or validating a tool and 127 studies used a cognitive assessment tool. Among the validation studies, measures of validity, reliability, or normative data were available for 22 cognitive assessment tools. 21 studies focused on criterion validity with 18 testing tools compared to clinical assessments and 3 compared to other scales. Cut‐off scores, sensitivity, and specificity were tested in 21 studies for a total of 15 tools. 35% of the validation studies had no reliability measures. Three studies developed a new assessment tool. The Mini Mental State Examination was the most frequently validated and used instrument across diverse settings. Studies validating domain‐specific tools were limited, with memory being the most frequently validated. Among studies using a cognitive tool in Arabic, over half used unvalidated versions.

**Conclusion:**

Psychometric evaluations of cognitive assessments for older Arabic‐speaking adults remain scarce with limited data regarding domain‐specific assessments, reproducibility, and suitability in different settings (different countries or different samples: clinical, community, nursing home). Findings emphasize this important challenge that can hinder robust data on ADRD burden and risk trajectories in Arab populations.

